# Analysis of Metabolic Subnetworks by Flux Cone Projection

**DOI:** 10.1186/1748-7188-7-17

**Published:** 2012-05-29

**Authors:** Sayed-Amir Marashi, Laszlo David, Alexander Bockmayr

**Affiliations:** 1 International Max Planck Research School for Computational Biology and Scientic Computing (IMPRS-CBSC), Max Planck Institute for Molecular Genetics, Ihnestr. 63-73, D-14195 Berlin, Germany; 2 FB Mathematik und Informatik, Freie Universität Berlin, Arnimallee 6, D-14195 Berlin, Germany; 3 DFG-Research Center Matheon, Berlin, Germany; 4 Berlin Mathematical School (BMS), Berlin, Germany

## Abstract

**Background:**

Analysis of elementary modes (EMs) is proven to be a powerful constraint-based method in the study of metabolic networks. However, enumeration of EMs is a hard computational task. Additionally, due to their large number, EMs cannot be simply used as an input for subsequent analysis. One possibility is to limit the analysis to a subset of interesting reactions. However, analysing an isolated subnetwork can result in finding incorrect EMs which are not part of any steady-state flux distribution of the original network. The ideal set to describe the reaction activity in a subnetwork would be the set of all EMs projected to the reactions of interest. Recently, the concept of "elementary flux patterns" (EFPs) has been proposed. Each EFP is a subset of the support (i.e., non-zero elements) of at least one EM.

**Results:**

We introduce the concept of ProCEMs (Projected Cone Elementary Modes). The ProCEM set can be computed by projecting the flux cone onto a lower-dimensional subspace and enumerating the extreme rays of the projected cone. In contrast to EFPs, ProCEMs are not merely a set of reactions, but projected EMs. We additionally prove that the set of EFPs is included in the set of ProCEM supports. Finally, ProCEMs and EFPs are compared for studying substructures of biological networks.

**Conclusions:**

We introduce the concept of ProCEMs and recommend its use for the analysis of substructures of metabolic networks for which the set of EMs cannot be computed.

## Background

Metabolic pathway analysis is the study of meaningful minimal pathways or routes of connected reactions in metabolic network models [1,2]. Two closely related concepts are often used for explaining such pathways: elementary modes (EMs) [3,4] and extreme pathways (EXPAs) [5]. Mathematically speaking, EMs and EXPAs are generating sets of the flux cone [1,6]. Several approaches have been proposed for the computation of such pathways [7-14].

EM and EXPA analysis are promising approaches for studying metabolic networks [15,16]. However, due to the combinatorial explosion of the number of such pathways [17,18], this kind of analysis cannot be performed for "large" networks. Recent advances in the computation of EMs and extreme rays of polyhedral cones [12,13] have made it possible to compute tens of millions of EMs, but computing all EMs for large genome-scale networks may still be impossible. Additionally, one is often interested only in a subset of reactions, and not all of them. Therefore, even if the EMs are computable, possibly many of them are not relevant because they are not related to the reactions of interest.

The goal of the present paper is to introduce the new concept of *Projected Cone Elementary Modes *(ProCEMs) for the analysis of substructures of metabolic networks. The organisation is as follows. Firstly, the mathematical concepts used in the text are formally defined. Secondly, we review the studies which have tried to investigate (some of) the EMs or EXPAs of large-scale networks. In the next step, we present the concept of ProCEMs and propose a method to compute them. Finally, we compare ProCEMs with elementary flux patterns (EFPs) from the mathematical and computational point of view, and analyse some concrete biological networks.

## Formal Definitions

We consider a metabolic network *N *with *m *internal metabolites and *n *reactions. Formally, we describe *N *by its stoichiometric matrix *S *∈ ℝ*^m × n ^*and the set of irreversible reactions *Irr *⊆ {1, . . ., *n*}. If steady-state conditions hold, i.e., there is no net production or consumption of internal metabolites, the set of all feasible flux distributions defines a polyhedral cone

(1) $$C=\left\{v\in {\mathbb{R}}^{n}|S\cdot v=0,v_{i}\geq 0\;\text{for}\;\text{all }i\in Irr\right\},$$

which is called the (steady-state) *flux cone *[1,2].

A *polyhedral cone in canonical form *is any set of the form *P *= {*x *∈ ℝ*^n ^| Ax *≤ 0}, for some matrix *A *∈ ℝ*^k × n^*. To bring (1) in canonical form, we can replace the equalities *Sv *= 0 by the two sets of inequalities *S *· *v *≤ 0 and *-S *· *v *≤ 0. Furthermore, the inequalities *v_i _*≥ 0, *i *∈ *Irr *are multiplied by -1. Any non-zero element *x *∈ *P *is called a *ray *of *P*. Two rays *r *and *r*' are *equivalent*, written *r *≅ *r*', if *r *= *λr*', for some *λ *> 0. A ray *r *in *P *is *extreme *if there do not exist rays *r*', *r*"∈ *P*, *r*' *≇ r*" such that *r *= *r*' + *r*".

For every *v *∈ ℝ*^n^*, the set *supp*(*v*) = {*i *∈ {1, . . ., *n*} | *v_i _*≠ 0 } is called the *support *of *v*.

A flux vector *e *∈ *C *is called an *elementary mode *(EM) [3,4] if there is no vector *v *∈ *C *\ {0} such that *supp*(*v*) ⊊ *supp*(*e*). Thus, each EM represents a minimal set of reactions that can work together in steady-state.

The set of all pairwise non-equivalent EMs, *E *= {*e*^1^, *e*^2^, . . ., *e^s^*}, generates the cone *C *[3]. This means that every flux vector in *C *can be written as a non-negative linear combination of the vectors in *E*.

Given a set *Q *⊆ *× *, where  resp.  are subspaces of ℝ*^n ^*of dimension *p *resp. *q *with *p *+ *q *= *n*, the *projection *of *Q *onto  is defined as

(2) $$P_{X}\left(Q\right)=\left\{x\in X|\exists y\in Y,\left(x,y\right)\in Q\right\}.$$

In the special case *Q *= {*v*}, we simply write $P_{X}\left(v\right)$ instead of $P_{X}\left(\left\{v\right\}\right)$.

Now consider a metabolic network  with *p *+ *q *reactions and a subnetwork $N^{\prime }$ given by a subset of *p *"interesting" reactions. For the flux cone *C *of  we assume C⊆X×Y, where the reactions of $N^{\prime }$ correspond to the subspace . The projection $P_{X}\left(C\right)$ of the cone *C *on the subspace  is again a polyhedral cone, called the *projected cone *on . Any elementary mode of the projected cone $P_{X}\left(C\right)$ will be called a *projected cone elementary mode *(ProCEM). The projection $P_{X}\left(e\right)$ of an elementary mode *e *∈ *C *to the subspace  will be called a *projected elementary mode *(PEM). As we will see in the sequel, the two concepts of PEM and ProCEM are closely related but different.

If the subnetwork $N^{\prime }$ has to be analysed, PEMs might be more relevant than EMs, as they are in lower dimension and easier to study. However, the only method currently known to compute PEMs is to enumerate the complete set of EMs and then to project these onto the subspace of interest. As we will see, ProCEMs provide an interesting alternative in this situation.

## The State of the Art

As mentioned above, the set of EMs of a genome-scale network may be large, and in general, cannot be computed with the available tools. Even if this is possible, one cannot simply extract interesting information from it. Therefore, a subset of EMs (or in case that we are interested in a subset of reactions, the set of PEMs) should be computed to reduce the running time and/or output size of the algorithm. Several approaches to this problem have been proposed in the literature. These strategies can be classified into four main categories:

### Computation of a Subset of EMs

The first strategy is to constrain the complete set of EMs (or EXPAs) to a subset describing a phenotype space or a set of phenotypic data. For example, Covert and Palsson [19] showed that consideration of regulatory constraints in the analysis of a small "core metabolism" model can reduce the set of 80 EXPAs to a set of 2 to 26 EXPAs, depending on the applied regulatory constraints. On the other hand, Urbanczik [20] suggested to compute "constrained" elementary modes which satisfy certain optimality criteria. As a result, instead of a full enumeration of EMs, only a subset of them should be computed, which results in a big computational gain. The idea of reducing the set of EMs has been used recently in an approach called *yield analysis *[21]. In this approach, the yield space (or solution space) is defined as a bounded convex hull. Then, the minimal generating set spanning the yield space is recalculated, and therefore, all EMs with negligible contribution to the yield space can be excluded. The authors show that their method results in 91% reduction of the EM set for glucose/xylose-fermenting yeast.

### Computation of EMs in Isolated Subsystems

A second strategy to focus on the EMs (or EXPAs) of interest is to select a (possibly disconnected) subsystem, rather than the complete metabolic model, by assuming all other reactions and metabolites to be "external", and computing the EMs (or EXPAs) of this selected subsystem. This idea, i.e., cutting out subsystems or splitting big networks into several subsystems, is broadly used in the literature (e.g., see [22-34]). In some of these studies, not only the network boundary is redrawn, but also some reactions may be removed for further simplifying the network.

Although this strategy is useful, it can result in serious errors in the computational analysis of network properties [35]. For example, dependencies and coupling relationships between reactions can be influenced by redrawing the system boundaries [36]. Burgard et al. [37] showed that subsystem-based flux coupling analysis of the *H. pylori *network [25] results in an incomplete detection of coupled reactions. Kaleta et al. [35] suggest that neglecting such a coupling can lead to fluxes which are not part of any feasible EM in the original complete network. Existence of such infeasible "pathway fragments" [38] can result in incorrect conclusions.

**Figure 1:**
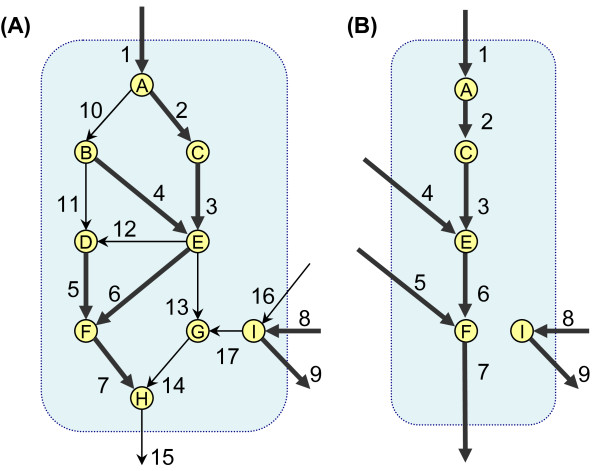
**An example metabolic subnetwork**. (A): A small metabolic network with 17 reactions. Metabolites are shown as nodes, while reactions are shown by arrows. Reactions 1, 8, 9, 15 and 16 are boundary reactions, while all other reactions are internal reactions. We might be interested only in a subnetwork containing nine reactions: 1, . . ., 9, which are shown by thick arrows. This subnetwork will be called SuN. (B): The reduced subsystem comprising only the nine interesting reactions.

To better understand this problem, we consider Figure 1A as an example. Let us assume that we are interested in a subnetwork composed of reactions 1, . . ., 9. This subnetwork is called SuN. If we simply assume the "uninteresting" reactions and metabolites to be the external reactions and metabolites, we will obtain the subsystem shown in Figure 1B. This subnetwork has only four EMs, two of which are not part of any feasible steady-state flux vector in the complete network. For example, the EM composed of reactions 5 and 7 in Figure 1B cannot appear in steady-state in the original complete network, because the coupling between reaction 1 and reaction 5 is broken. Therefore, analyzing this subnetwork instead of the original network can result in false conclusions.

### Computation of Elementary Flux Patterns

We observed that some errors may appear in the analysis of isolated subsystems. One possible solution to this problem is to compute a "large" subset of PEMs, or alternatively, as suggested by Kaleta et al. [35], to compute the support of a subset of PEMs. These authors proposed a procedure to compute the elementary flux patterns (EFPs) of a subnetwork within a genome-scale network. A *flux pattern *is defined as a set of reactions in a subnetwork that is included in the support of some steady-state flux vector of the entire network [35]. A flux pattern is called an *elementary flux pattern *if it cannot be generated by combination of two or more different flux patterns. Each EFP is the support of (at least) one PEM. It is suggested that in many applications, the set of EFPs can be used instead of EMs [35].

Although EFPs are promising tools for the analysis of metabolic pathways, they also have their own shortcomings. The first important drawback of EFPs is that they cannot be used in place of EMs in certain applications [9], where the precise flux values are required. For example, in the identification of all pathways with optimal yield [23,39] and in the analysis of control-effective fluxes [27,28,40], the flux values of the respective reactions in the EMs should be taken into account.

Another important shortcoming of EFP analysis is that it is possible to have very different EMs represented by the same EFP, since flux values are ignored in EFPs. For example, consider the case that two reactions *i *and *j *are partially coupled [37]. This means that there exist at least two EMs, say *e *and *f*, such that *e_i_*/*e_j _*≠ *f_i_/f_j _*[41]. However, if we consider a subnetwork composed of these two reactions, then we will only have one EFP, namely {*i*, *j*}. From the theoretical point of view, finding all EMs that correspond to a certain EFP is computationally hard (see Theorem 2.7 in [42]).

Every EFP is related to at least one EM in the original metabolic network. However, one of the limitations of EFP analysis is that EFPs are activity patterns of some EMs, not necessarily all of them. We will show this by an example. In Figure 1A, the flux cone is a subset of ℝ^17^, while the subnetwork SuN induces a 9-dimensional subspace $X={\mathbb{R}}^{9}$. If *G *is the set of EMs in Figure 1A, then the set of PEMs can be computed as $P=\left\{P_{X}\left(e\right)|e\in G\right\}$. The set of the 10 PEMs of SuN in Figure 1A is shown in Table 1. For the same network and subnetwork, we used EFPTools [43] to compute the set of the EFPs. The resulting 7 EFPs are also presented in Table 1. If we compare the PEMs and EFPs, we find out that the support of each of the first 7 PEMs is equal to one of the EFPs. However, for the last three PEMs no corresponding EFP can be found in Table 1. This is due to the fact that *supp*(*p*8) = *E*4 ∪ *E*5, *supp*(*p*9) = *E*3 ∪ *E*5, and *supp*(*p*10) = *E*1 ∪ *E*2. Hence, the flux patterns corresponding to these PEMs are not elementary. Therefore, some EMs may exist in the network which have no corresponding EFP on a certain subnetwork. This means that by EFP analysis possibly many EMs of the original network cannot be recovered. Informally speaking, we ask whether the set of EFPs is the largest set of PEM supports which can be computed without enumerating all EMs.

**Table 1:** List of elementary flux patterns, projected cone elementary modes and projected elementary modes of SuN.

EFPs	EFP set	ProCEM	PEM	vector
*E*1	{9}	*u*1	*p*1	(0, 0, 0, 0, 0, 0, 0, 0, 1)
*E*2	{8}	*u*2	*p*2	(0, 0, 0, 0, 0, 0, 0, 1, 0)
*E*3	{1, 4}	*u*3	*p*3	(1, 0, 0, 1, 0, 0, 0, 0, 0)
*E*4	{1, 2, 3}	*u*4	*p*4	(1, 1, 1, 0, 0, 0, 0, 0, 0)
*E*5	{1, 5, 7}	*u*5	*p*5	(1, 0, 0, 0, 1, 0, 1, 0, 0)
*E*6	{1, 4, 6, 7}	*u*6	*p*6	(1, 0, 0, 1, 0, 1, 1, 0, 0)
*E*7	{1, 2, 3, 6, 7}	*u*7	*p*7	(1, 1, 1, 0, 0, 1, 1, 0, 0)
-	-	*u*8	*p*8	(1, 1, 1, 0, 1, 0, 1, 0, 0)
-	-	*u*9	*p*9	(1, 0, 0, 1, 1, 0, 1, 0, 0)
-	-	-	*p*10	(0, 0, 0, 0, 0, 0, 0, 1, 1)

Flux through reactions 1, . . ., 9, respectively, are the elements of the shown vectors. Zero vector and also the empty set are excluded.

### Projection Methods

**Figure 2:**
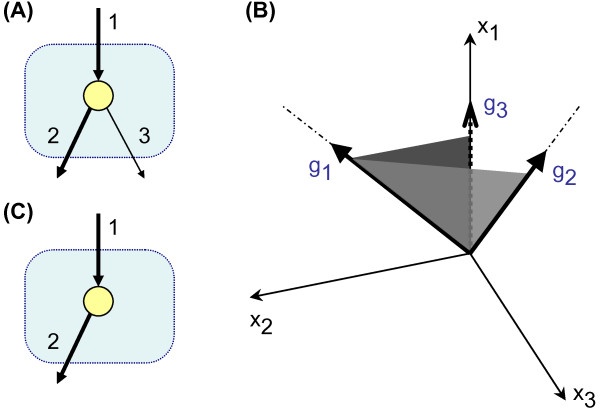
**Flux cone projection**. (A): A small metabolic network. The reactions in the interesting subnetwork are shown as thick arrows. (B): The flux cone of this network, shown in light gray, can be generated by vectors *g*_1 _and *g*_2_. The projected cone is shown in dark gray. The projected cone can be generated by *g*_1 _and *g*_3 _in a 2D plane. (C): the same metabolic network as in A, but with reaction 3 removed. The flux cone of this network is generated by only one vector, namely *g*_1_.

A possible strategy to simplify the network analysis is to project the flux cone down to a lower-dimensional space of interest. In other words, if we are interested in a subnetwork, we may project the flux cone onto the lower-dimensional subspace defined by the "interesting" reactions. Note that projecting the flux cone is in general different from removing reactions from the network. Consider the simple network shown in Figure 2A and a graphical representation of its corresponding flux space in Figure 2B (here, the axes *x*_1_, *x*_2_, *x*_3 _correspond to reactions 1, 2, 3, thus the flux cone is the open triangle shown in light gray). This network has two EMs, which are the generating vectors of the flux cone, *g*_1 _and *g*_2_. Now, if we are interested in a subnetwork composed of reactions 1 and 2, then we can project the flux cone to the 2D subspace produced by these two reactions. This is comparable to light projection on a 3D object to make 2D shadows. The projected cone is shown in dark gray. When the flux cone is projected onto the lower-dimensional space, new generating vectors may appear. In this example, *g*_1 _and *g*_3 _(in 2D space) are the generating vectors of the projected cone. Intuitively, one can think about *g*_3 _as the projected flux vector through reaction 1 and 3. This projected flux cone is certainly different from the flux cone of a network made by deleting reaction 3 (Figure 2C). Such a network has only one EM, and its corresponding flux cone can be generated by only one vector, namely, *g*_1_.

Historically, the idea of flux cone projection has already been used in some papers. Wiback and Palsson [44] suggested that the space of cofactor production of red blood cell can be studied by projecting the cell-scale metabolic network onto a 2D subspace corresponding to ATP and NADPH production. A similar approach was used by Covert et al. [19] and also by Wagner and Urbanczik [45] to analyze the relationship between carbon uptake, oxygen uptake and biomass production. All the above studies considered very small networks. Therefore, the authors computed the extreme rays of the flux cone and then projected them onto the subspace of interest, without really projecting the flux cone. Urbanczik and Wagner [46] later introduced the concept of *elementary conversion modes *(ECMs), which are in principle the extreme rays of the cone obtained by projecting the original flux cone onto the subspace of boundary reactions. They suggest that the extreme rays of this "conversion" cone, i.e., the ECMs, can be computed even for large networks [47].

Following this idea, we introduce the ProCEM set ("Projected Cone Elementary Mode" set), which is the set of EMs of the projected flux cone. In contrast to [46], we formulate the problem in a way that any subnetwork can be chosen, not only the boundary reactions. Additionally, we compare the closely related concepts of ProCEMs, PEMs and EFPs.

## Method and Implementation

### Computational Procedure

Our algorithm needs three input objects: the stoichiometric matrix *S *∈ ℝ*^m×n ^*of the network *is *, the set of irreversible reactions *Irr *⊆ {1, . . ., *n*}, and the set of reactions ∑ ⊆ {1, . . ., *n*} in the subnetwork of interest, while as an output it will return the complete set of ProCEMs. The computation of ProCEMs is achieved in three main consecutive steps.

**Step 1 - Preprocessing: **The aim of this step is to remove inconsistencies from the metabolic network and to transform it into a form suitable for the projection in Step 2. First, based on ∑ we sort the columns of *S *in the form:

(3) $$\bar{S}=\left(Ā\;\bar{B}\right)$$

where the reaction corresponding to the *i*-th column belongs to ∑ iff the *i*-th column is in *Ā*. Next, the blocked reactions [37] are removed. Finally, each of the reversible reactions is split into two irreversible "forward" and "backward" reactions. The final stoichiometric matrix will be in the form:

(4) $$S^{\prime }=\left(A\;B\right)$$

where the columns of *A *represent the "interesting" reactions after splitting reversible reactions and removing the blocked reactions. In the following, we assume that *A *(resp. *B*) has *p *(resp. *q*) columns. Given *S*', the steady-state flux cone in canonical form will look as follows

(5) $$C=\left\{\left(x,y\right)\in {\mathbb{R}}^{p+q}|G\cdot x+H\cdot y\leq 0\right\},$$

where matrix *G *(resp. *H*) represent the columns to be kept (resp. eliminated):

(6) $$G=\left(\begin{array}{c} -A\\ A\\ -I_{p}\\ 0_{q,p} \end{array}\right),\;H=\left(\begin{array}{c} -B\\ B\\ 0_{p,q}\\ -I_{q} \end{array}\right)$$

Here *I_p _*denotes the *p *× *p *identity matrix, and 0*_p,q _*the *p *× *q *zero matrix.

**Step 2 - Cone Projection: **In this step, the flux cone is projected, eliminating the reactions corresponding to columns in *H*. Several methods have been proposed in the literature for the projection of polyhedra [48]. For our purpose we chose the *block elimination method *[49]. This method allows us to find an inequality description of the projected cone by enumerating the extreme rays of an intermediary cone called the *projection cone*. In our case, the projection cone is defined as

(7) $$W=\left\{w\in {\mathbb{R}}^{2m+p+q}|H^{T}\cdot w=0,w\geq 0\right\},$$

where *H^T ^*denotes the transpose of *H*.

We enumerate the extreme rays {*r*^1^, *r*^2^, . . ., *r^k^*} of *W *using the double description method [50]. The projected cone is given by

(8) $$P_{X}\left(C\right)=\left\{x\in {\mathbb{R}}^{p}|R\cdot G\cdot x\leq 0\right\},$$

where

(9) $$R={\left(r^{1}...r^{k}\right)}^{T}.$$

This representation of the projected cone contains as many inequalities as there are extreme rays in *W*, thus a large number of them might be redundant [48]. These redundant inequalities are removed next (see below).

**Step 3 - Finding ProCEMs: **In the final step, the extreme rays of the projected cone, i.e., the ProCEMs, are enumerated. Similarly as in Step 2, the double description method is employed to enumerate the extreme rays of $P_{X}\left(C\right)$.

With the block elimination algorithm, it is also possible to perform the projection in an iterative manner. This means that rather than eliminating all the "uninteresting" reactions in one step, we can partition these in *t *subsets and then iteratively execute Step 2, eliminating every subset of reactions one by one. By proceeding in this fashion, the intermediary projection cones, *W*^1^, *W*^2^, . . ., *W^t ^*get typically smaller, thus enumerating their extreme rays requires less memory. On the other side, the more sets we partition into, the slower the projection algorithm usually gets.

### Implementation and Computational Experiments

The ProCEM enumeration algorithm has been implemented in MATLAB v7.5. In our implementation, polco tool v4.7.1 [12,13] is used for the enumeration of extreme rays (both in Step 2 and 3). For removing redundant inequalities in Step 2, the redund method from the lrslib package v4.2 is used [51]. All computations are performed on a 64-bit Debian Linux system with Intel Core 2 Duo 3.0 GHz processor. A prototype implementation is available on request from the authors.

### Dataset

The metabolic network model of red blood cell (RBC) [44] is used in this study. The network is taken from the example metabolic networks associated with CellNetAnalyzer [52] and differs slightly from the original model. Additionally, we studied the plastid metabolic network of *Arabidopsis thaliana *[53] (see Additional file 1). Then, the subnetwork of "sugar and starch metabolism" is selected as the interesting subnetwork of the plastid metabolic network.

## Results and Discussion

### Mathematical Relationships among PEMs, EFPs and ProCEMs

From Table 1, one can observe that the set of ProCEMs in Figure 1A is included in the set of PEMs. Additionally, the set of EFPs is included in the set of ProCEM supports. Here, we prove that these two properties are true in general. This means that the analysis of ProCEMs has at least two advantages compared to the analysis of EFPs. Firstly, ProCEMs can tell us about the flux ratio of different reactions in an elementary mode, while EFPs can only tell us whether the reaction has a non-zero value in that mode. Secondly, enumeration of ProCEMs may result in modes which cannot be obtained by EFP analysis.

**Theorem 1**. *In a metabolic network  with irreversible reactions only, let J (resp. P) be the set of ProCEMs (resp. PEMs) for a given set of interesting reactions. Then J *⊆ *P*.

*Proof*. We have to show that for every *u *∈ *J *there exists an elementary mode *e *∈ *C *in  such that $P_{X}\left(e\right)≅u$. We know that for any *u *∈ *J *there exists *v *∈ *C *such that $P_{X}\left(v\right)=u$.

Any *v *∈ *C *can be written in the form $v=\sum_{k=1}^{r}c_{k}\cdot e^{k}$, where *e*^1^, . . ., *e^r ^*are elementary modes of  and *c*_1_, . . ., *c_r _>*0. It follows that $P_{X}\left(v\right)=\overset{r}{\underset{k=1}{\sum }}c_{k}\cdot P_{X}\left(e^{k}\right)$.

If all the vectors $P_{X}\left(e^{k}\right)$ are pairwise equivalent, *u *is a PEM.

Otherwise, *u *is a linear combination of at least two non-equivalent PEMs, which are vectors in $P_{X}\left(C\right)$.

This implies that *u *is not an extreme ray of $P_{X}\left(C\right)$, in contradiction with Lemma 1 in [9] saying that in a metabolic network with irreversible reactions only, the EMs are exactly the extreme rays. □

**Theorem 2**. *In a metabolic network  with irreversible reactions only, let E (resp. J) be the set of EFPs (resp. ProCEMs) for a given set of interesting reactions. Then*, *E *⊆ {*supp*(*u*) | *u *∈ *J*}.

*Proof*. Suppose that for some *F *∈ *E*, there exists no *v *∈ *J *such that *F *= *supp*(*v*). Since *F *is an EFP, there exists *p *∈ *P *such that *F *= *supp*(*p*). It follows *p *∉ *J*, but $p\in P_{X}\left(C\right)$, where *C *is the flux cone. Therefore, there exist *r *≥ 2 different ProCEMs, say *u*^1^,..., *u^r ^*∈ *J*, such that $p=\overset{r}{\underset{k=1}{\sum }}c_{k}\cdot u^{k}$, with *c_k _>*0 for all *k*. Since *u^k ^*≥ 0, for all *k*, we have $supp\left(p\right)=\overset{r}{\underset{k=1}{⋃}}supp\left(u^{k}\right)$, with *supp*(*u^k^*) ≠ *supp*(*p*) for all *k*. Since *supp*(*u^k^*) is a flux pattern for all *k*, this is a contradiction with *F *being an EFP. □

### Computing the Set of EFPs from the Set of ProCEMs

Here, we present a simple algorithm to show that it is possible to compute the set of EFPs when the set of ProCEMs is known. Table 2 summarizes this procedure.

**Table 2:** Algorithm 1: Computing the set of EFPs based on the set of ProCEMs

**Input**:
• *J *(the set of ProCEMs)
**Output**:
• *E *(the set of EFPs)
**Initialization:**
*E *:= ∅;
**Main procedure:**


We know that the support of every ProCEM *u *is a flux pattern *Z*. In the main procedure, we check whether every such flux pattern is elementary or not. If *Z *is *not *elementary, then it is equal to the union of some other flux patterns. Therefore, if all other flux patterns which are subsets of *supp*(*u*) are subtracted from *Z*, this set becomes empty. This algorithm has the complexity $O\left(nq^{2}\right)$, where *q *is the number of ProCEMs and *n *is the number of reactions.

### Comparing EFPs and ProCEMs

#### Analysis of Subnetworks in the Metabolic Network of RBC

In order to compare our approach (computation of ProCEMs) with the enumeration of EFPs, we tested these methods for analysing subnetworks of the RBC model [44]. Again, we split every reversible reaction into one forward and one backward irreversible reaction. The resulting network contains 67 reactions, including 20 boundary reactions, and a total number of 811 EMs. For comparing the methods, the set of all boundary reactions was considered as the interesting subsystem, resulting in 502 PEMs.

When we computed the EFPs of this network by EFPTools [43], only 90 EFPs are determined. However, for the same subnetwork, we computed 252 ProCEMs. This means that the ProCEMs set covers more than half of the PEMs, while the EFPs set covers less than one fifth of the PEMs. These results confirm the relevance of using ProCEMs for the analysis of subnetworks.

**Figure 3:**
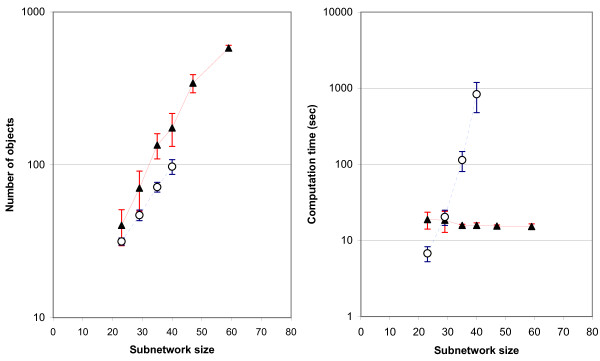
**ProCEM vs. EFP computation**. Left: Number of ProCEMs and EFPs computed for random subnetworks of different sizes. Right: The computation times (per second) required for computing the ProCEMs and EFPs in the left chart. ▲: ProCEMs; O: EFPs. Each experiment is repeated 100 times. Confidence intervals in this plot are based on one-sample *t*-test (95% c.i.). For large subnetworks (*r *> 40), we did not compute the EFPs because the program was very slow.

In order to compare the computation of EFPs and ProCEMs, the following task was performed on the RBC model [44]. In each iteration, a random subnetwork containing *r *reactions was selected. Then, EFPs and ProCEMs were computed. The task was repeated for different subnetwork sizes. The computational results can be found in Figure 3.

From Figure 3, it can be seen that EFP computation is faster than ProCEM computation for small subnetworks. However, when the subnetwork size *r *increases, computation of ProCEMs does not become slower, while computation of EFPs significantly slows down. This is an important observation, because the difference between the number of EFPs and ProCEMs also increases with *r*.

#### Analysis of Subnetworks in the Plastid Metabolic Network of A. *thaliana*

ProCEM analysis becomes important when PEMs cannot be computed. This may happen frequently in the analysis of large-scale metabolic networks, as memory consumption is a major challenge in computation of EMs [12]. In such cases, cone projection might still be feasible.

As an example, the metabolic network of *A. thaliana *plastid was studied (Additional file 1). This network contains 102 metabolites and 123 reactions (205 reactions after splitting reversible reactions). Using efmtool (and also polco) [12], even after specifying 2 GB of memory, computation of EMs was not possible due to running out of memory. Therefore, for no subnetwork of the plastid network, PEMs could be computed. However, if the analysis is restricted to the 57 reactions involved in sugar and starch metabolism (see Additional file 1), one can compute the ProCEMs or EFPs of this subnetwork. We computed the ProCEMs as described in the Method and Implementation section, using a projection step size of 5 reactions. The complete set of 1310 ProCEMs was computed in approximately 15 minutes. However, when we tried to compute the set of EFPs using EFPTools [35,43], only 279 EFPs were computed after 4 days of running the program (270 EFPs were computed in the first two days). On the other hand, using a Matlab implementation of Algorithm 1, the complete set of 1054 EFPs was obtained in 30 seconds. In conclusion, in metabolic networks for which the set of EMs cannot be enumerated, ProCEMs prove to be a useful concept to get insight into reaction activities.

## Conclusions

In this paper, we introduce the concept of projected cone elementary modes (ProCEMs). The set of ProCEMs covers more PEMs than EFPs. Therefore, ProCEMs contain more information than EFPs. The set of ProCEMs is computable without enumerating all EMs. Is there a bigger set of vectors that covers even more PEMs and does not require full enumeration of EMs? This question is yet to be answered. One possible extension to this work is to use a more efficient implementation of polyhedral projection. With such an implementation, analysis of different subnetworks in genome-scale network models using ProCEMs is an interesting possibility for further research. For example, the ProCEMs can be used in the identification of all pathways with optimal yield [23] and in the analysis of control-effective fluxes [27].

## Competing interests

The authors declare that they have no competing interests.

## Authors' contributions

The original idea was presented by SAM, LD and AB. The mathematical results are presented by SAM, and improved by all authors. Implementation of the ProCEM method and performing the computational experiments are done by LD. The manuscript was originally drafted by SAM, and improved by all authors. The final version of the manuscript was read and approved by all authors.

## Supplementary Material

Additional file 1 **Plastid metabolic network**. In the first tab of this Excel file, general information about the plastid network of *A. thaliana *is mentioned. In the second tab, stoichiometric matrix and the set of reversible reactions (as a binary vector) is included. In the third tab, the reactions involved in sugar and starch metabolism are listed. Click here for file
